# Author Correction: An algorithm to quantify intratumor heterogeneity based on alterations of gene expression profiles

**DOI:** 10.1038/s42003-022-03285-0

**Published:** 2022-03-31

**Authors:** Mengyuan Li, Zhilan Zhang, Lin Li, Xiaosheng Wang

**Affiliations:** 1 Biomedical Informatics Research Lab, School of Basic Medicine and Clinical Pharmacy, China Pharmaceutical University, Nanjing, 211198 China; 2 Cancer Genomics Research Center, School of Basic Medicine and Clinical Pharmacy, China Pharmaceutical University, Nanjing, 211198 China; 3 Big Data Research Institute, China Pharmaceutical University, Nanjing, 211198 China

**Keywords:** Cancer, Tumour heterogeneity, Computational biology and bioinformatics, Tumour heterogeneity

Correction to: *Communications Biology* 10.1038/s42003-020-01230-7, published online 11 September 2020.

The original version of the Article contained errors in the Algorithm formulas in the Methods section. The formula

$$	\frac{1}{m-1}\sqrt{\mathop{\sum }\limits_{i=1}^{m}{\left({\left(ex({G}_{i},TS)-\frac{1}{n}\mathop{\sum }\limits_{j=1}^{n}ex({G}_{i},N{S}_{j})\right)}^{2}-\frac{1}{m}\mathop{\sum }\limits_{i=1}^{m}{\left(ex({G}_{i},TS)-\frac{1}{n}\mathop{\sum }\limits_{j=1}^{n}ex({G}_{i},N{S}_{j})\right)}^{2}\right)}^{2}}\\ 	 =\frac{1}{m-1}\sqrt{\mathop{\sum }\limits_{i=1}^{m}{\left(\varDelta {({G}_{i},TS,N{S}_{j})}^{2}-\frac{1}{m}\mathop{\sum }\limits_{i=1}^{m}\varDelta {({G}_{i},TS,N{S}_{j})}^{2}\right)}^{2}},\varDelta ({G}_{i},TS,N{S}_{j}) \\ 	=ex({G}_{i},TS)-\frac{1}{n}\mathop{\sum }\limits_{j=1}^{n}ex({G}_{i},N{S}_{j}),$$

has been replaced with

$$	\sqrt{\frac{1}{m-1}\left(\mathop{\sum }\limits_{i=1}^{m}{\left({\left(ex({G}_{i},TS)-\frac{1}{n}\mathop{\sum }\limits_{j=1}^{n}ex({G}_{i},N{S}_{j})\right)}^{2}-\frac{1}{m}\mathop{\sum }\limits_{i=1}^{m}{\left(ex({G}_{i},TS)-\frac{1}{n}\mathop{\sum }\limits_{j=1}^{n}ex({G}_{i},N{S}_{j})\right)}^{2}\right)}^{2}\right)}\\ 	= \,\sqrt{\frac{1}{m-1}\left(\mathop{\sum }\limits_{i=1}^{m}{\left(\varDelta {({G}_{i},TS,N{S}_{j})}^{2}-\frac{1}{m}\mathop{\sum }\limits_{i=1}^{m}\varDelta {({G}_{i},TS,N{S}_{j})}^{2}\right)}^{2}\right)},\varDelta ({G}_{i},TS,N{S}_{j})\\ 	= \,ex({G}_{i},TS)-\frac{1}{n}\mathop{\sum }\limits_{j=1}^{n}ex({G}_{i},N{S}_{j}),$$

and the formula

$$	\frac{1}{m-1}\sqrt{\mathop{\sum }\limits_{i=1}^{m}{\left({\left(ex({G}_{i},TS)-\frac{1}{t}\mathop{\sum }\limits_{j=1}^{t}ex({G}_{i},C{S}_{j})\right)}^{2}-\frac{1}{m}\mathop{\sum }\limits_{i=1}^{m}{\left(ex({G}_{i},TS)-\frac{1}{t}\mathop{\sum }\limits_{j=1}^{t}ex({G}_{i},C{S}_{j})\right)}^{2}\right)}^{2}} \\ 	= \,\frac{1}{m-1}\sqrt{\mathop{\sum }\limits_{i=1}^{m}{\left(\varDelta {({G}_{i},TS,C{S}_{j})}^{2}-\frac{1}{m}\mathop{\sum }\limits_{i=1}^{m}\varDelta ({G}_{i},TS,C{S}_{j})\right)}^{2},}\varDelta ({G}_{i},TS,C{S}_{j})= \,ex({G}_{i},TS)-\frac{1}{t}\mathop{\sum }\limits_{j=1}^{t}ex({G}_{i},C{S}_{j}),$$

has been replaced with

$$	\sqrt{\frac{1}{m-1}\left(\mathop{\sum }\limits_{i=1}^{m}{\left({\left(ex({G}_{i},TS)-\frac{1}{t}\mathop{\sum }\limits_{j=1}^{t}ex({G}_{i},C{S}_{j})\right)}^{2}-\frac{1}{m}\mathop{\sum }\limits_{i=1}^{m}{\left(ex({G}_{i},TS)-\frac{1}{t}\mathop{\sum }\limits_{j=1}^{t}ex({G}_{i},C{S}_{j})\right)}^{2}\right)}^{2}\right)} \\ 	= \,\sqrt{\frac{1}{m-1}\left(\mathop{\sum }\limits_{i=1}^{m}{\left(\varDelta {({G}_{i},TS,C{S}_{j})}^{2}-\frac{1}{m}\mathop{\sum }\limits_{i=1}^{m}\varDelta {({G}_{i},TS,C{S}_{j})}^{2}\right)}^{2}\right)}, \varDelta ({G}_{i},TS,C{S}_{j}) = \,ex({G}_{i},TS)-\frac{1}{t}\mathop{\sum }\limits_{j=1}^{t}ex({G}_{i},C{S}_{j}),$$

In addition, the sentence “The DEPTH algorithm (R package) is available are available at Github (https://github.com/WangX-Lab/DEPTH).”

has been replaced by

“The DEPTH algorithm (R function) is available at https://github.com/WangX-Lab/DEPTH/ under a GNU GPL open-source license.”.

This has now been corrected in the PDF and HTML versions of the Article.

