# Supplementary material for: An algorithm to quantify intratumor heterogeneity based on alterations of gene expression profiles
Source: Commun Biol. 2020 Sep 11;3:505. doi: 10.1038/s42003-020-01230-7 (PMC7486929; doi:10.1038/s42003-020-01230-7)
Supplement: Supplementary file 1 — Supplmentary Information [file 42003_2020_1230_MOESM1_ESM.pdf]

Supplementary Information

Supplementary Figure 1. Association of DEPTH scores with clinical characteristics in individual cancer types. (a) Kaplan−Meier survival curves showing the negative correlation between DEPTH scores and overall survival (OS) in multiple cancer types. The log-rank test *p*-values, hazard ratio (HR), and 95% confidence interval (CI) are shown. high-DEPTH-score, DEPTH scores in the upper third. low-DEPTH-score, DEPTH scores in the bottom third. (**b)** Kaplan−Meier survival curves showing the negative correlation between DEPTH scores and OS trends in both MSI and non-MSI COAD. (**c)** DEPTH scores increase with the advancement of substage in pan-cancer.

Supplementary Figure 2. DEPTH scores are negatively associated with the survival (OS, DSS, DFI, and PFI) in pan-cancer and 3 individual cancer types (BRCA, COAD, and KIRC) after correcting the tumor-infiltrating lymphocyte (TIL) level (Cox proportional hazards test, $p < 0.02$).

Supplementary Figure 3. 9 genes whose somatic mutations are associated with increased DEPTH scores in at least 5 cancer types and are associated with worse overall survival in pan-cancer. Kaplan−Meier survival curves showing the survival time differences between gene-mutated and gene-wildtype tumors. The log-rank test *p*-values, HR, and 95% CI are shown.

Supplementary Figure 4. Logistic regression analysis showing that DEPTH scores have a significant negative correlation with antitumor immune signatures in most cases after correcting tumor purity.

Supplementary Figure 5. Comparison of DEPTH with other ITH evaluation methods. (a-e) Comparisons of the correlations of genomic instable features (a), clinical features **(b)**, immune signatures **(c)**, drug response **(d)**, and tumor purity **(e)** with ITH scores from seven different methods (DEPTH, tITH [24], sITH [26], EXPANDS [4,5], PhyloWGS [6], ABSOLUTE [2], and MATH [3]). HRD, homologous recombination deficiency. **(f)** Comparison of DEPTH with tITH in the validation datasets.

Supplementary Figure 6. DEPTH score distribution within individual cancer types.

Supplementary Figure 7. ITH score distribution for tITH, sITH, EXPANDS, PhyloWGS, MATH, and ABSOLUTE within individual cancer types.

Supplementary Data 1. Correlations between DEPTH scores and drug sensitivity (IC50 values) of 265 compounds tested in cancer cell lines.

Supplementary Data 2. 262 genes whose expression alteration has a strong positive correlation with DEPTH scores in at least 5 cancer types (FDR < 0.05, $\rho > 0.5$)

Supplementary Data 3. Proteins with significantly higher (or lower) expression levels in high-DEPTH-score than in low-DEPTH-score tumors in at least 5 cancer types.

Supplementary Data 4. Clone numbers inferred by DEPTH across cancer types.

Supplementary Data 5. Correlations between ITH scores from seven different methods within individual cancer types.

Supplementary Data 6. Association between ITH scores from seven different methods and tumor purity within individual cancer types.

Supplementary Data 7. Correlations of the DEPTH scores calculated by the alternative method (without normal control) with genome instability, clinical features, immune signatures, and tumor purity.

Supplementary Data 8. A summary of the gene expression profiling datasets used in this study.

Supplementary Data 9. The marker genes of immune signatures, proliferation, and tumor stemness.

Supplementary Note 1. Comparisons of DEPTH with other ITH evaluation methods.

Supplementary Figures

Supplementary Figure 1

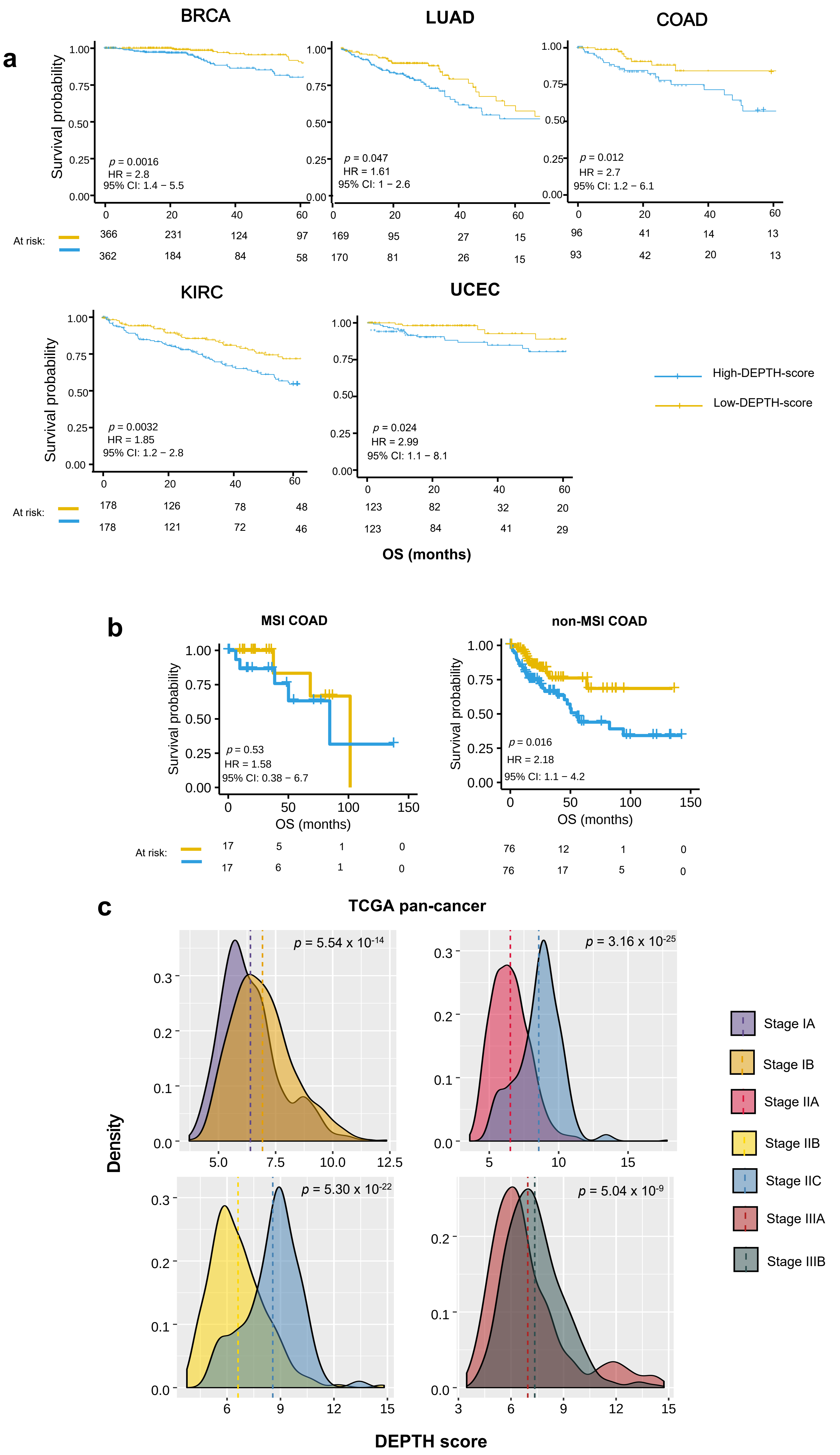

Supplementary Figure 2

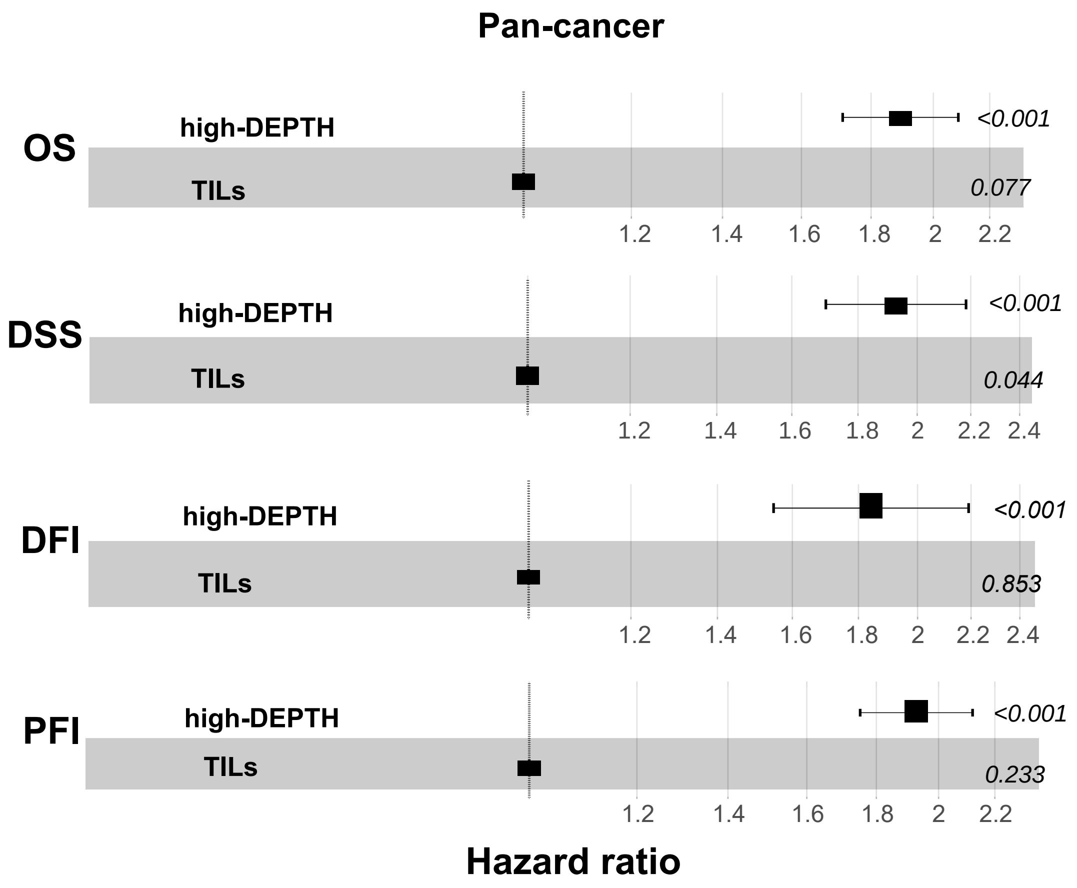

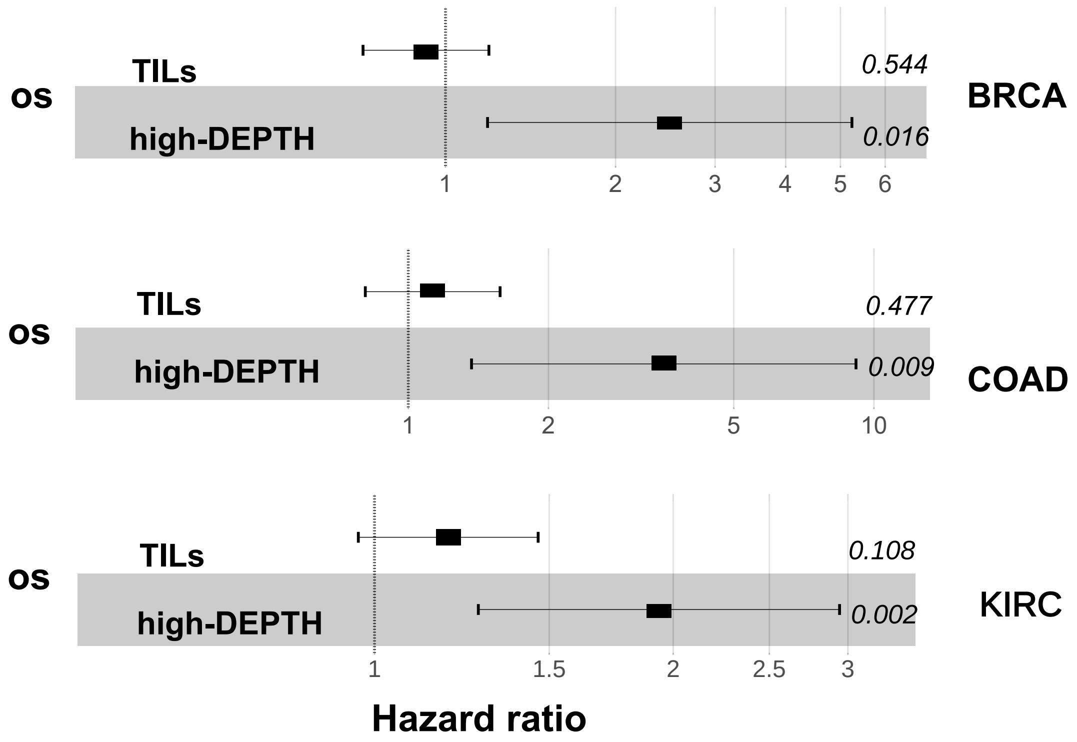

Supplementary Figure 3

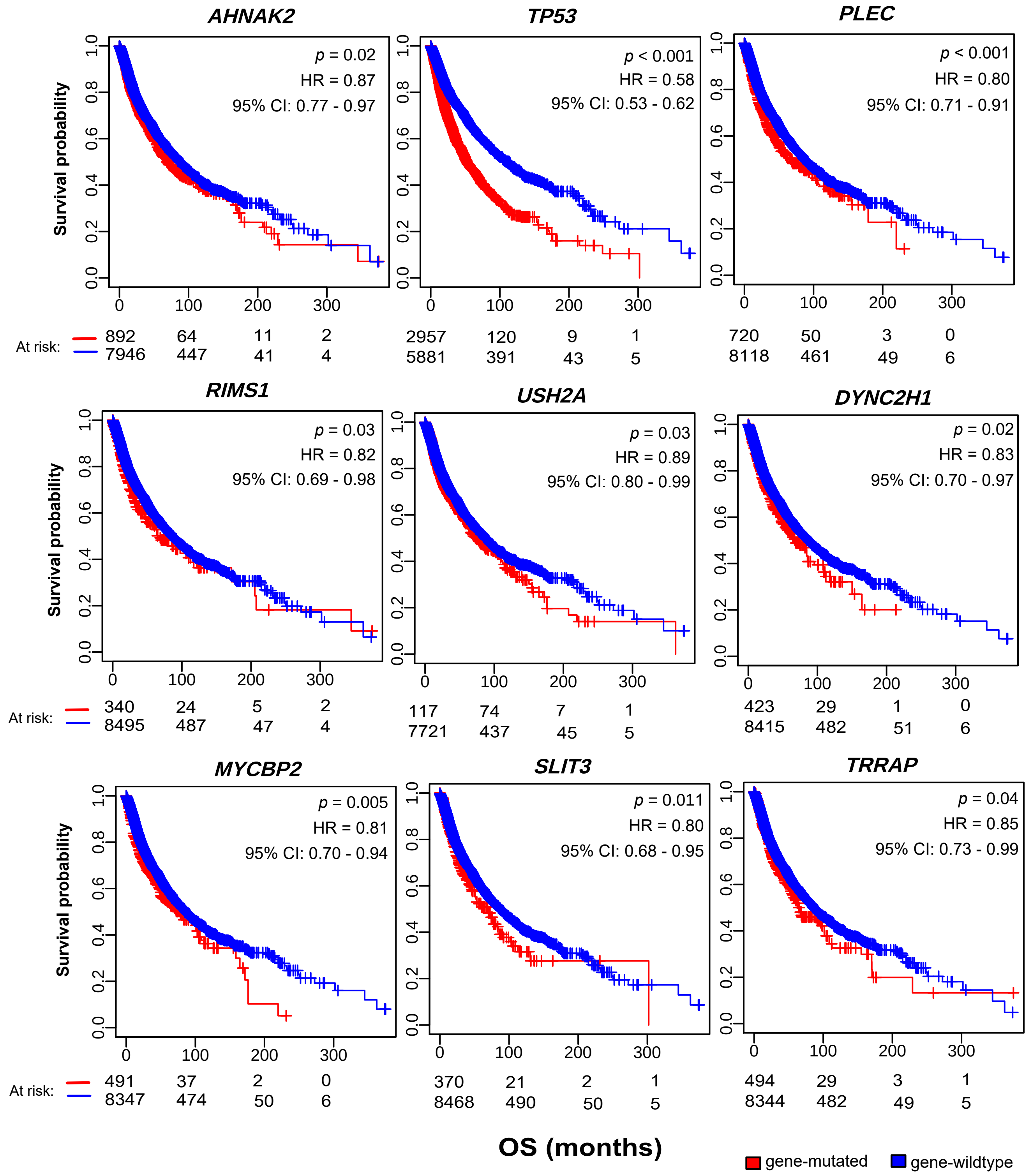

Supplementary Figure 4

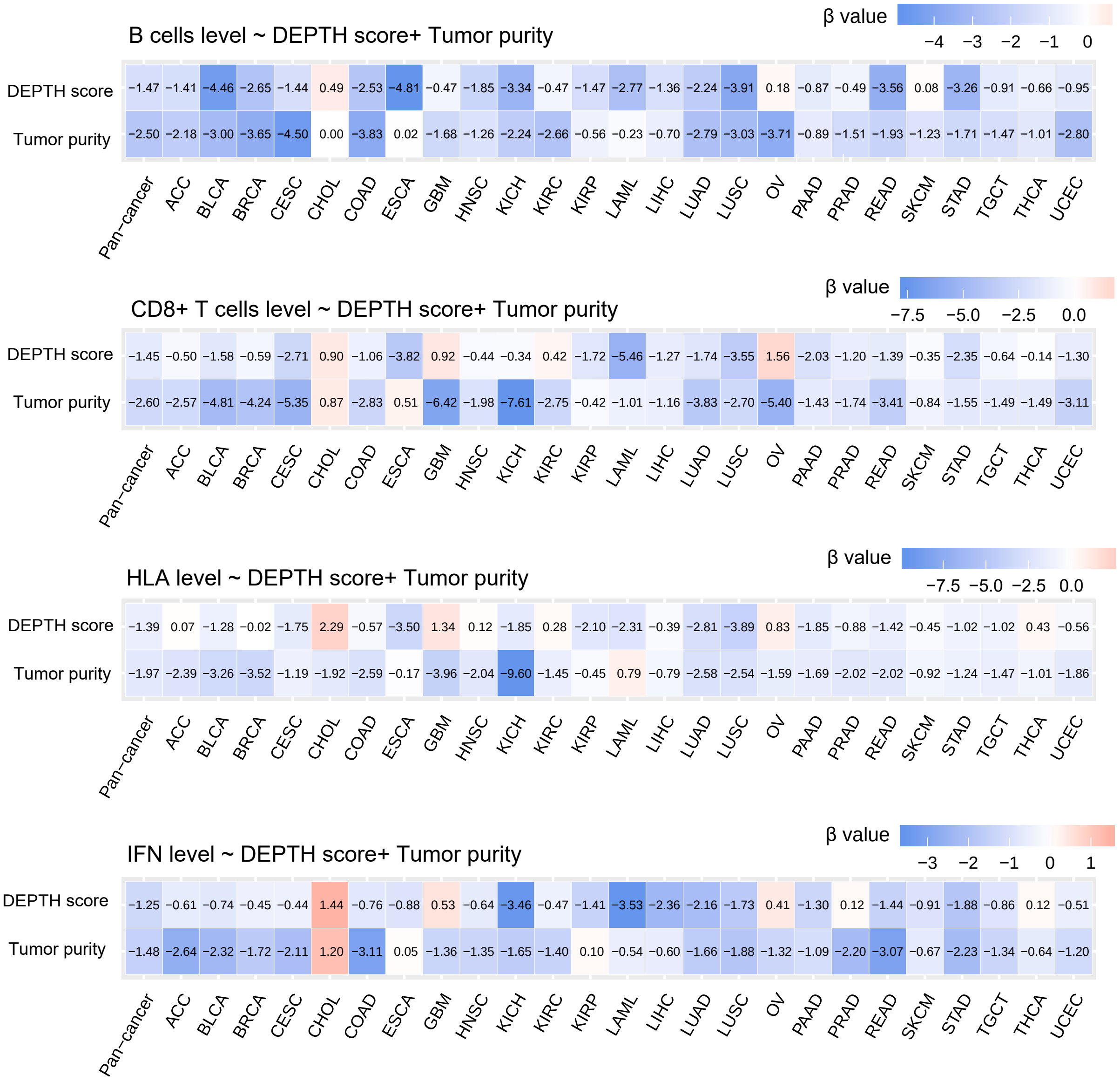

Supplementary Figure 5

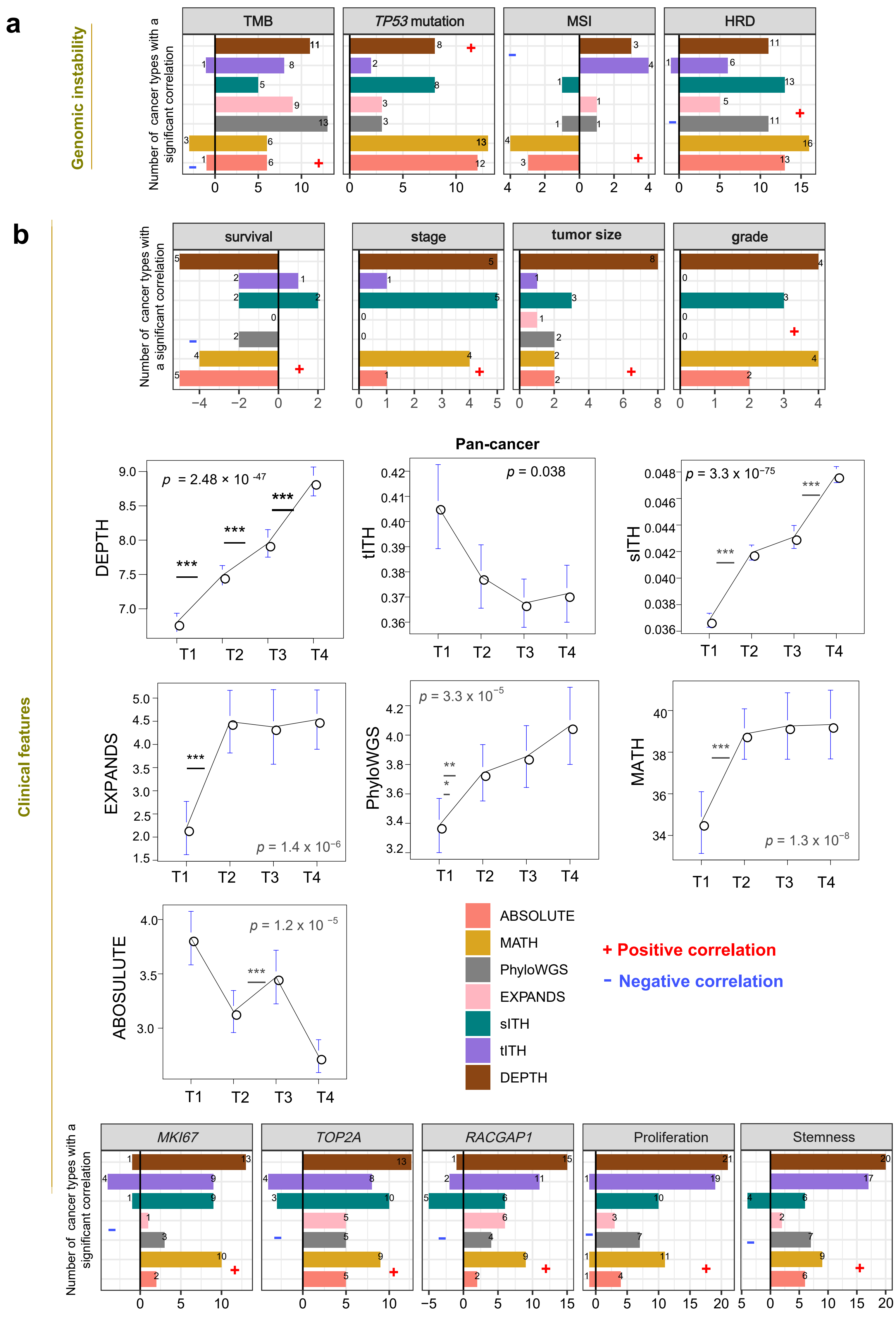

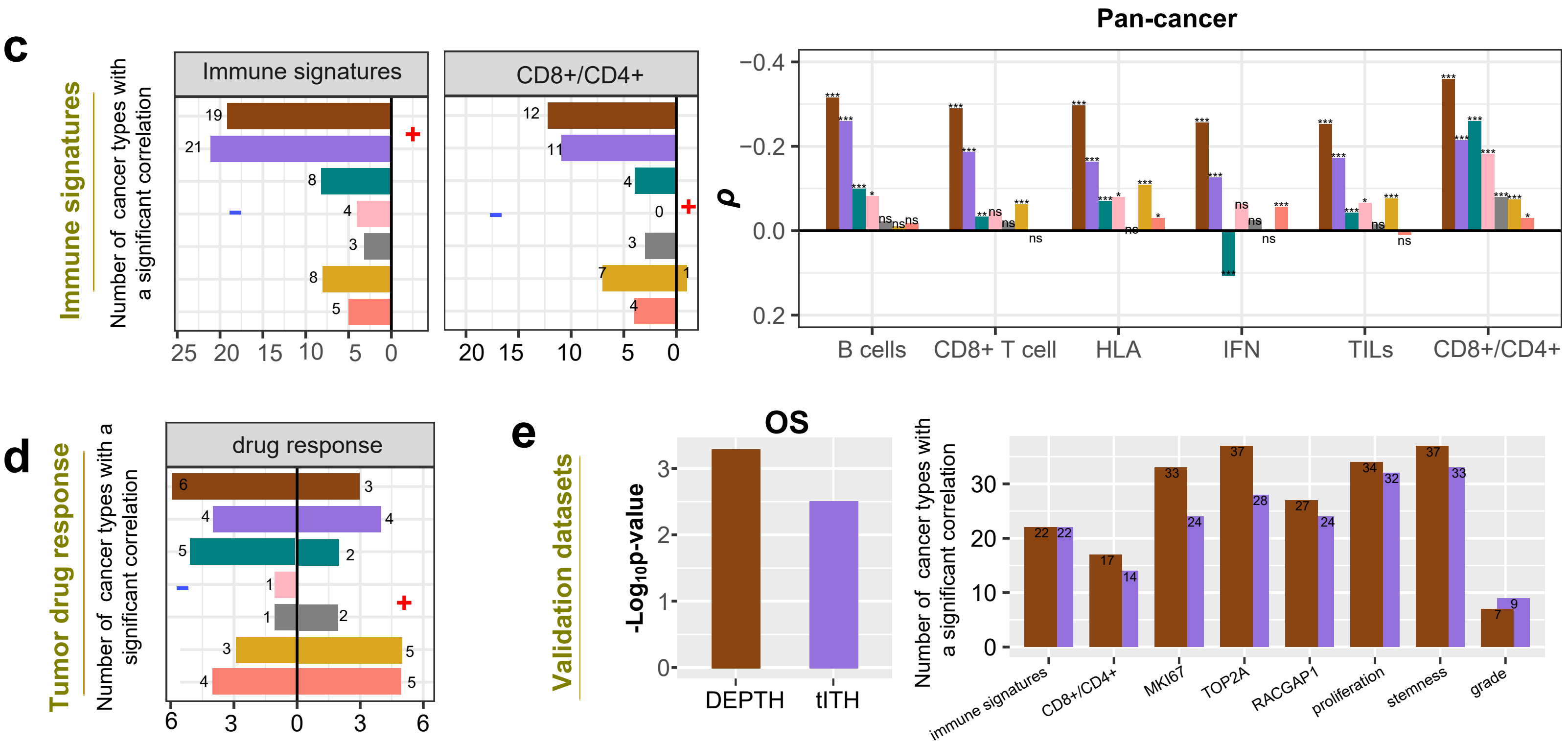

c
Immune signatures
Number of cancer types with a significant correlation
Immune signatures
CD8+/CD4+
19
21
8
4
3
8
5
12
11
4
0
3
7
1
4
25 20 15 10 5 0
20 15 10 5 0
Pan-cancer
ρ
−0.4
−0.2
0.0
0.2
B cells
CD8+ T cell
HLA
IFN
TILs
CD8+/CD4+
d
Tumor drug response
Number of cancer types with a significant correlation
drug response
6 3
4 4
5 2
1
1 2
3 5
4 5
6 3 0 3 6
e
Validation datasets
OS
-Log10p-value
DEPTH
tITH
Number of cancer types with a significant correlation
22 22
17 14
33 24
37 28
27 24
34 32
37 33
7 9
immune signatures
CD8+/CD4+
MKI67
TOP2A
RACGAP1
proliferation
stemness
grade

Supplementary Figure 6

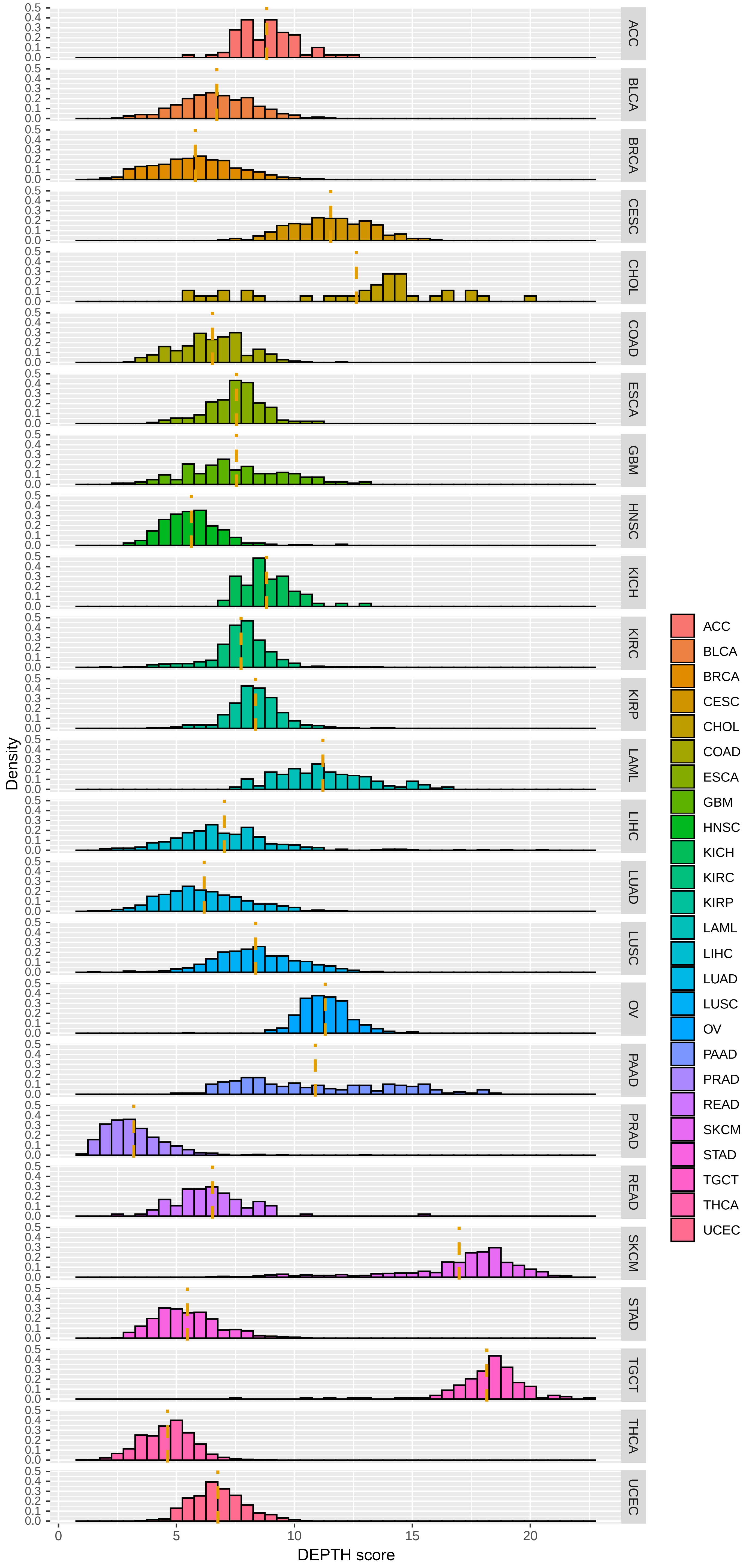

Supplementary Figure 7

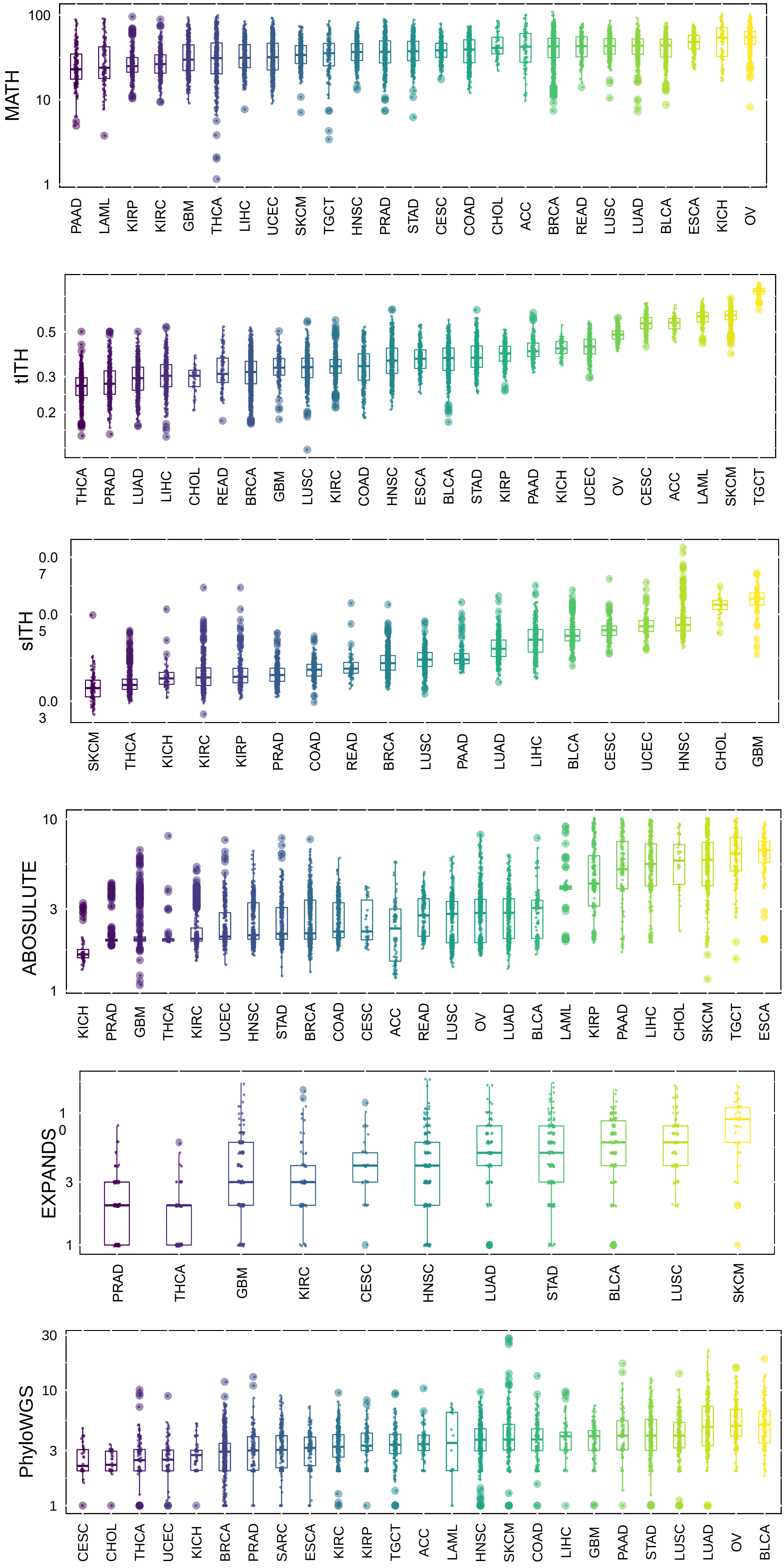

Supplementary Note 1

Comparisons of DEPTH with other ITH evaluation methods

We compared DEPTH with other ITH evaluation methods in their correlations with genomic instability, clinical features, antitumor immune signatures, tumor drug response, and tumor purity. Moreover, we compared DEPTH with tITH using the validation datasets.

1. Genomic instability

TMB positively correlated with ITH scores from tITH, sITH, EXPANDS, PhyloWGS, MATH, and ABSOLUTE in 8, 5, 9, 13, 6, and 6 cancer types, respectively, compared to DEPTH scores in 11 cancer types ($FDR_{sp} < 0.05$) (Supplementary Figure 5a); however, TMB had a negative correlation with ITH scores from tITH, MATH, and ABSOLUTE in 1, 3, and 1 cancer types, respectively. ITH scores from tITH, sITH, EXPANDS, PhyloWGS, MATH, and ABSOLUTE were significantly higher in *TP53*-mutated than in *TP53*-wildtype tumors in 2, 8, 3, 3, 13, and 12 individual cancer types, respectively, compared to DEPTH scores in 8 cancer types ($FDR < 0.05$) (Supplementary Figure 5a). In the five cancer types (COAD, ESCA, READ, STAD, and UCEC) encompassing a high proportion of MSI-H tumors, ITH scores from tITH, EXPANDS, and PhyloWGS were significantly higher in MSI-H than in MSI-L/MSS tumors in 4, 1 and 1 individual cancer types, respectively, compared to DEPTH scores in 3 cancer types ($p < 0.05$); however, ITH scores from sITH, PhyloWGS, MATH, and ABSOLUTE were lower in MSI-H than in MSI-L/MSS tumors in 1, 1, 4, and 3 individual cancer types, respectively (Supplementary Figure 5a). Furthermore, ITH scores from tITH, sITH, EXPANDS, PhyloWGS, MATH, and ABSOLUTE positively correlated with HRD scores in 6, 13, 5,

11, 16, and 13 individual cancer types, respectively, compared to DEPTH scores in 11 cancer types (FDR < 0.05) (Supplementary Figure 5a).

Overall, these results indicate that DEPTH scores have comparable positive correlations with the genomic instability features compared to the other ITH scores. Notably, compared to another transcriptome-based method (tITH), DEPTH scores displayed a stronger and more consistent correlation with TMB, *TP53* mutations, and HRD scores in more cancer types.

2. Clinical features

Like DEPTH scores, ITH scores from tITH, sITH, PhyloWGS, and MATH had negative correlations with four survival endpoints (OS, DSS, DFI, and PFI) in pan-cancer (log-rank test, $p < 0.05$). ITH scores from EXPANDS and ABSOLUTE showed negative correlations with three (OS, DFI, and PFI) and two survival endpoints (DFI and PFI), respectively. Furthermore, ITH scores from tITH, sITH, EXPANDS, PhyloWGS, MATH, and ABSOLUTE inversely correlated with OS in 2 (BRCA and LUAD), 2 (COAD and KIRC), 0, 2 (ACC and PAAD), 4 (ACC, COAD, ESCA, and UCEC), and 5 (ACC, CHOL, KIRP, HNSC, and UCEC) cancer types, respectively, compared to DEPTH scores in 5 cancer types (BRCA, COAD, KIRC, LUAD, and UCEC) (log-rank test, $p < 0.05$) (Supplementary Figure 5b). However, unlike the mRNA-based methods, most of the cancer types in which the negative correlation was observed between ITH scores from the DNA-based methods and OS are not prevalent. Unexpectedly, ITH scores from two RNA-Seq-based methods (tITH and sITH) displayed a significant positive correlation

with OS in 1 (LUSC) and 2 (HNSC and UCEC) cancer types, respectively. Overall, these results indicate that DEPTH scores display a stronger negative correlation with OS than the DNA-based ITH scores in the prevalent cancer cohorts, including BRCA, LUAD, COAD, and KIRC. Meanwhile, compared to the other two mRNA-based methods (tITH and sITH), DEPTH scores are more likely to be a consistent prognostic factor across individual cancer types.

ITH scores from tITH, sITH, EXPANDS, PhyloWGS, MATH, and ABSOLUTE were significantly higher in late-stage than in early-stage tumors in 1, 5, 0, 0, 4, and 1 cancer types, respectively, compared to DEPTH scores in 5 cancer types, and were significantly higher in T3-4 than in T1-2 tumors in 1, 3, 1, 2, 2, and 2 cancer types, respectively, compared to DEPTH scores in 8 cancer types (FDR < 0.05) (Supplementary Figure 5b). Also, ITH scores from tITH, sITH, EXPANDS, PhyloWGS, MATH, and ABSOLUTE were significantly higher in high-grade than in low-grade tumors in 0, 3, 0, 0, 4, and 2 cancer types, respectively, compared to DEPTH scores in 4 cancer types (FDR < 0.05) (Supplementary Figure 5b). Collectively, these results indicate that DEPTH scores have a stronger correlation with tumor advancement than most of the other ITH scores. In particular, DEPTH scores positively correlated with tumor size in many more cancer types versus all the other ITH scores. Moreover, DEPTH scores consistently and significantly increased across T1, T2, T3, and T4 in pan-cancer (one-sided Kruskal–Wallis test, $p = 2.5 \times 10^{-47}$) but we did not observe such a trend in the other ITH scores (Supplementary Figure 5b). Again, DEPTH scores displayed a more significant correlation with tumor advancement than tITH scores (Supplementary Figure 5b).

The significant positive correlation between the expression of three proliferation markers (*MKI67*, *TOP2A*, and *RACGAP1*) and ITH scores was more prevalent in DEPTH than in the other ITH scores in individual cancer types ($FDR_{SP} < 0.05$) (Supplementary Figure 5b). Moreover, the enrichment levels of the proliferation signature positively correlated with ITH scores from tITH, sITH, EXPANDS, PhyloWGS, MATH, and ABSOLUTE in 19, 10, 3, 7, 11, and 4 cancer types, respectively, compared to DEPTH scores in 21 cancer types ($FDR_{SP} < 0.05$) (Supplementary Figure 5b). Furthermore, DEPTH scores displayed a more prevalent positive correlation with tumor stemness scores than the other ITH scores (Supplementary Figure 5b); ITH scores from tITH and sITH showed a positive correlation with tumor stemness scores in 17 and 6 cancer types, respectively, compared to DEPTH scores in 20 cancer types ($FDR_{SP} < 0.05$); ITH scores from sITH displayed a significant negative correlation with tumor stemness scores in 4 cancer types, while such negative correlation was not observed in the other ITH scores (Supplementary Figure 5b).

Taken together, these results indicate that DEPTH scores have a more prevalent and consistent positive correlation with unfavorable clinical outcomes than the other ITH scores.

3. Antitumor immune signatures

Likewise, we analyzed the correlations of ITH scores from the other six methods with the five antitumor immune signatures in TCGA cancers. In general, these ITH scores were

likely to have a negative correlation with these immune signatures (Supplementary Figure 5c). However, DEPTH scores showed stronger correlations than the other ITH scores in both pan-cancer and individual cancer types. In pan-cancer, the correlation coefficients of the other ITH scores with the immune signatures ranged in [-0.26, 0.11], compared to DEPTH scores in [-0.31, -0.25] (Supplementary Figure 5c). Moreover, DEPTH scores showed a significant negative correlation with the immune signatures in much more individual cancer types than the other ITH scores (except tITH scores) ($FDR_{SP} < 0.05$). In 19 individual cancer types, DEPTH scores had significant inverse correlations with at least 3 of the 5 immune signatures, compared to the other ITH scores in 21 (tITH), 8 (sITH), 4 (EXPANDS), 3 (PhyloWGS), 8 (MATH), and 5 (ABSOLUTE) cancer types, respectively (Supplementary Figure 5c). Besides, ITH scores from the other six methods were also inversely associated with the ratios of CD8+ T cells/CD4+ regulatory T cells in pan-cancer ($-0.21 \leq \rho \leq -0.08$), compared to DEPTH scores with ρ = -0.31 (Supplementary Figure 5c). ITH scores from tITH, sITH, EXPANDS, PhyloWGS, MATH, and ABSOLUTE were inversely associated with the ratios of CD8+ T cells/CD4+ regulatory T cells in 11, 4, 0, 3, 7, and 4 cancer types, respectively, compared to DEPTH scores in 12 cancer types ($FDR_{SP} < 0.05$).

Overall, the two transcriptome-based ITH scores (DEPTH and tITH) show more significant correlations with antitumor immune signatures than the other ITH scores.

4. Tumor drug response

We compared ITH scores between responsive and non-responsive groups in TCGA datasets. When all drugs combined, like DEPTH scores, the ITH scores from tITH, PhyloWGS, and ABSOLUTE were significantly higher in the non-responsive group than in the responsive group in pan-cancer (p = 0.022, 0.0015, 0.0003, respectively). Nevertheless, the differences in DEPTH scores between both groups appear to be more significant (p = 0.0001). In contrast, MATH scores were significantly higher in the responsive group than in the non-responsive group (p = 0.016). The ITH scores from sITH and EXPANDS showed no significant differences between both groups ($p > 0.1$). Furthermore, in 4, 5, 1, 1, 3, and 4 individual drugs, respectively, the ITH scores from tITH, sITH, PhyloWGS, EXPANDS, MATH, and ABSOLUTE were significantly higher in the non-responsive group than in the responsive group in pan-cancer ($p < 0.05$), as compared to DEPTH scores in 6 drugs (Supplementary Figure 5d). On the other hand, in 4, 2, 0, 2, 5, and 5 individual drugs, respectively, the ITH scores from tITH, sITH, PhyloWGS, EXPANDS, MATH, and ABSOLUTE were significantly lower in the non-responsive group than in the responsive group in pan-cancer ($p < 0.05$), as compared to DEPTH scores in 3 drugs. Collectively, these results indicate that the ITH defined by DEPTH is more likely to capture the association between tumor heterogeneity and drug resistance than that defined by the other methods.

5. Tumor purity

Like DEPTH scores, the ITH scores from EXPANDS, tITH, and sITH were likely to be positively associated with tumor purity, as shown in 6, 17, and 6 cancer types, respectively ($FDR_{sp} < 0.05$) (Supplementary Data 6). In contrast, ABSOLUTE and

MATH scores were inversely associated with tumor purity in diverse cancer types (Supplementary Data 6), indicating that high ABSOLUTE and MATH scores are associated with low tumor purity. These results suggest that the mRNA-based ITH scores are more likely to capture the ITH in tumor cells than the DNA-based ITH scores (except EXPANDS), which are prone to be confounded by non-tumor components.

6. Comparison of DEPTH with tITH in the validation datasets

We compared the performance between DEPTH and tITH in the validation datasets. This comparison was restricted to 37 datasets containing normal samples since tITH only applies to the gene expression profiling data with normal controls. In the E-MTAB-6703 breast cancer dataset which contained both survival data and gene expression data in normal samples, tITH scores showed a significant inverse correlation with OS (log-rank test, $p = 0.003$), compared to a more significant inverse correlation of DEPTH scores with OS (log-rank test, $p = 0.0005$) (Supplementary Figure 5e). In 33 of the 37 datasets, tITH scores were significantly associated with tumor stemness scores ($p < 0.05$, $0.17 \leq \rho \leq 0.79$), compared to DEPTH scores in 37 datasets ($p < 0.05$, $0.10 \leq \rho \leq 0.92$). In addition, in 32 datasets, tITH scores had a significant positive correlation with proliferation signature ($p < 0.05$, $0.10 \leq \rho \leq 0.81$), compared to DEPTH scores in 34 datasets ($p < 0.05$, $0.23 \leq \rho \leq 0.83$). In 22 datasets, tITH scores had significant inverse correlations with at least 3 of the 5 immune signatures ($p < 0.05$), compared to DEPTH scores in 22 datasets, and in 14 datasets, tITH scores had significant inverse correlations with CD8+/CD4+ regulatory T cells ($p < 0.05$), compared to DEPTH scores in 17 datasets. In 11 datasets with the tumor grade phenotype data, tITH scores were markedly higher in high-grade

than in low-grade tumors in 9 datasets ($p < 0.05$), compared to DEPTH scores in 7 datasets. Collectively, these results revealed that tITH scores had weaker correlations with survival prognosis, tumor stemness, and proliferation signature, and comparable correlations with immune signatures and tumor advancement compared to DEPTH scores in the validation datasets.
