# Supplementary material for: An algorithm to quantify intratumor heterogeneity based on alterations of gene expression profiles
Source: Commun Biol. 2020 Sep 11;3:505. doi: 10.1038/s42003-020-01230-7 (PMC7486929; doi:10.1038/s42003-020-01230-7)
Supplement: Supplementary file 2 — Description of Additional Supplementary Files [file 42003_2020_1230_MOESM2_ESM.pdf]

Description of Additional Supplementary Files

File Name: Supplementary Data 1
Description: Correlations between DEPTH scores and drug sensitivity (IC50 values) of 265 compounds tested in cancer cell lines.

File Name: Supplementary Data 2
Description: 262 genes whose expression alteration has a strong positive correlation with DEPTH scores in at least 5 cancer types (FDR < 0.05, $\rho > 0.5$)

File Name: Supplementary Data 3
Description: Proteins with significantly higher (or lower) expression levels in high-DEPTH-score than in low-DEPTH-score tumors in at least 5 cancer types.

File Name: Supplementary Data 4
Description: Clone numbers inferred by DEPTH across cancer types.

File Name: Supplementary Data 5
Description: Correlations between ITH scores from seven different methods within individual cancer types.

File Name: Supplementary Data 6
Description: Association between ITH scores from seven different methods and tumor purity within individual cancer types.

File Name: Supplementary Data 7
Description: Correlations of the DEPTH scores calculated by the alternative method (without normal control) with genome instability, clinical features, immune signatures, and tumor purity.

File Name: Supplementary Data 8
Description: A summary of the gene expression profiling datasets used in this study.

File Name: Supplementary Data 9
Description: The marker genes of immune signatures, proliferation, and tumor stemness.
